# Contemporary Surgical Management of Severe Sialorrhea in Children 

**DOI:** 10.5402/2012/364875

**Published:** 2012-03-28

**Authors:** Jeremy Hornibrook, Neil Cochrane

**Affiliations:** Department of Otolaryngology, Head and Neck Surgery, Christchurch Hospital, 2 Riccarton Avenue, Christchurch 8011, New Zealand

## Abstract

The causes of severe sialorrhea (drooling) are reviewed, and in particular in children in whom it can become a life-long disability. The history of medical and surgical treatments is discussed. A major advance has been the surgical relocation of the submandibular gland ducts with removal of sublingual glands. The results of this operation, technical considerations, and its outcomes in 16 children are presented. There were no significant complications. Caregivers judged the efficacy with a median score of “75%” improvement. The technique has become the most logical and reliable surgical treatment for drooling, with very good control in most cases. In contrast to “Botox” its effects are permanent.

## 1. Introduction

**Figure 1:**
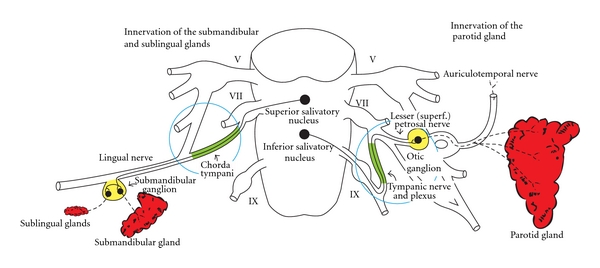
Parasympathetic innervation of the salivary glands. Otological access is to the chorda tympanic nerve for the submandibular and sublingual glands and the tympanic plexus for the parotid gland.

An adult produces about 1.2 litres of saliva a day, mainly from three pairs of major salivary glands and from minor glands in the oral cavity and palate. The relative contributions to volume are minor glands 5%, sublingual glands 5%, parotids 30%, and submandibular glands 70%. The parasympathetic nerve supply to the salivary glands is illustrated in Figure 1. Saliva lubricates food to assist in chewing, and the swallowing process contains digestive enzymes and has a role in dental health.

Sialorrhea (“drooling”, “dribbling”, and “drivelling”) is the involuntary escape of saliva from the mouth. In contrast to excess saliva production (salivation) it implies an inability to retain saliva due to lip incontinence, or to decreased oral sensation, a defective oral stage of swallowing, an open bite, poor posture and neck flexion, or a combination of those factors. Minor “normal” drooling is not uncommon in normal children (usually boys) up to the age of five. In contrast, pathological drooling is a major cause of poor quality of life in individuals with major neurological disability. Strokes, Parkinson's disease, and motor neurone disease can be a cause of sialorrhea in late adult life. Of greater significance is that drooling can be a major and potential life-long disability in physically and intellectually impaired children. Between 10% and 37% of children with cerebral palsy display pathological drooling [1]. The cause is not overproduction of saliva [2] but impaired lip control, causing a delay between the suction and propelling stages of the oral phase of swallowing [3]. Consequently, saliva from the sublingual and submandibular glands pools in the anterior floor of mouth and spills out. The spectrum of drooling severity ranges from minimally affected to severe and persistent when it can have major negative effects on the physical and social well-being of the individual as well as on their family, caregivers, and peers. The physical effects can be cracked lips, odour, and soaking of bedding, clothes toys, school books, caregivers, and peers. Frequent clothing and bib changes may be required. It can lead to social isolation and, in individuals with less cognitive disability, self-consciousness and depression. 

Attempts to treat pathological drooling can be summarised as behavioural (speech therapy, physiotherapy),  pharmacotherapy, and surgery. In some centres physiotherapists, occupational therapists and speech-language  therapists offer a combination of techniques such as posture/positioning, oral-motor therapy, and behaviour modification techniques to increase sensory awareness and voluntary swallowing. This approach is time-consuming, the relapse rate is high, and it only suits a small group of well-motivated subjects [4]. Traditional pharmacological treatment has been anticholinergic drugs to inhibit salivary production, either as atropine oral drops or scopolamine patches. Side effects are dry mouth, blurred vision, and urinary retention. The response rate is variable and unpredictable, and side effects are common.

The first surgical treatment for drooling was relocation of the parotid ducts [5], followed by the additional removal of the submandibular glands [6]. Later, even the more radical bilateral division of the parotids ducts and removal of the submandibular glands was advocated [7, 8], and more recently a “four-duct ligation” [9]. In the 1970s, neurectomies (division of the chorda tympani nerve and tympanic plexus in the middle ear) were attempted (Figure 1) [10–12]. These were mainly in adults, with equivocal efficacy, late relapse, and attracted the criticism that it entailed an operation on a normal ear with loss of taste sensation if both were divided treated [12].

 In 1974, Ekedahl from Scandinavia described a new operation [13] for sialorrhea—relocation of the submandibular ducts to the tonsillar fossae. 99 mTc isotope salivary gland uptake studies indicated functioning glands and the likely normal passage of saliva to the oropharynx. Its preservation of normal (but diverted) salivary function and its simplicity were appealing. Subsequently it has become (with refinements) the surgical technique of choice for severe drooling. The main advocate has been Crysdale in Canada [14–16], others in Australia [8, 17, 18], and elsewhere [19–21].

## 2. Methods

In a small series of children and teenagers with sialorrhea treated (1993–2011) by surgery, the indications and results were analysed from hospital inpatient, outpatient, and specific database records. Demographic details and causes are presented in Table 1. All had severe sialorrhea, requiring changes of clothes during the day, despite extra measures such as bibs and scarves. None had aspiration. One patient had had previous surgical treatment.

**Figure 2:**
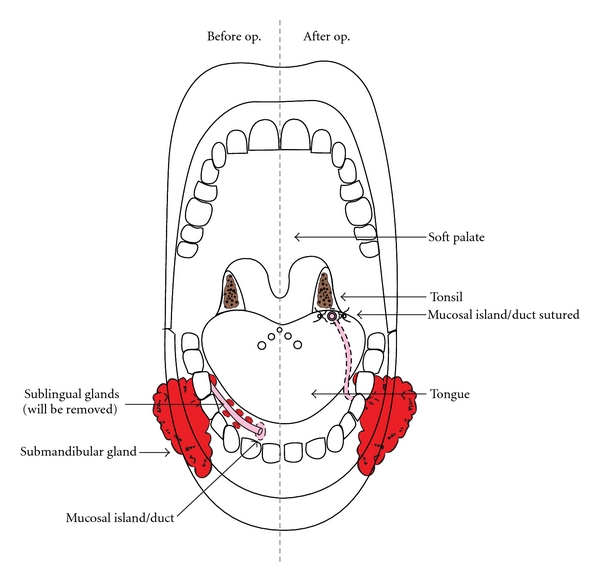
Contemporary drooling surgery: relocation of the submandibular ducts, with removal of the sublingual glands.

The operation is performed under general anaesthesia with nasal intubation to enhance access to the tongue and floor of mouth (Figure 2). All patients were given intravenous dexamethasone and antibiotics. If possible the submandibular duct is cannulated. A mucosal island around the duct opening is made, and the floor of mouth mucosa opened to allow dissection of the the duct to just above the submandibular gland. All sublingual glands are removed. A tunnel is made towards the tongue base emerging beside the anterior pillar. A vascular tape is passed through, the mucosal island and duct attached and then pulled through to the tongue base, where it is sutured. The floor of mouth incision is closed with a running absorbable suture. All steps of the operation are shown in the videoclip, see supplementary materials available at doi:10.5402/2012/364875 (Video 1). The surgical procedures and technical variations encountered are presented in Table 2. One patient had later removal of sublingual glands. At six weeks, care givers were asked to grade the efficacy on a linear scale: 0–25%, 25–50%, 50–75%, and 75–100%.

## 3. Results

**Figure 3:**
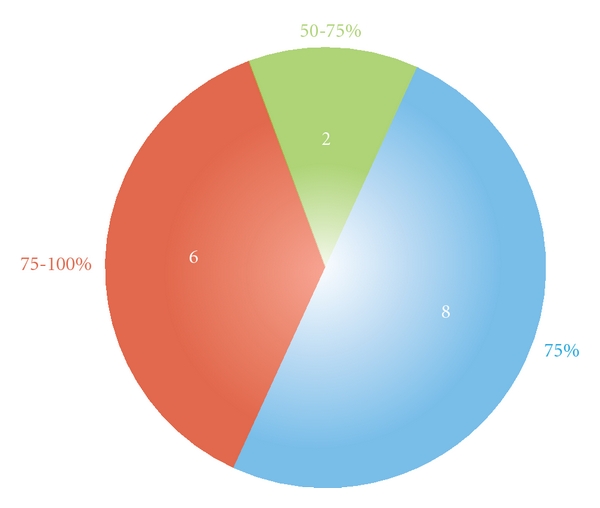
Final efficacy (percent improvement) as adjudged by caregivers of 16 children with severe sialorrhea treated surgically.

Details of hospital stay, complications and final efficacy are presented in Table 3. The median hospital stay was two nights. There were two minor and temporary complications. Only one patient was readmitted. The final efficacy (percentage improvement) median score was 75% (range 50%–100%) (Figure 3).

## 4. Discussion

For severe drooling, it is unlikely that behavioural techniques or traditional pharmacotherapy effective, or at least practical for a lifetime. The earliest surgical treatments were radical [5–8] with undesired consequence of painful swelling of salivary glands and a likely near total lack of saliva. For ethical and technical reasons, neurectomies have proved to be undesirable and unpredictable. Ekehdahl's [13] report of relocation of the submandibular ducts was an original and appealing advance—elimination of saliva pooling in the anterior floor of mouth, but insuring its diverted presence in the pharynx to assist the swallowing process, for life.

The earliest advocates of submandibular duct relocation in Canada and USA reported favourable drooling rates [15, 16, 22], but there were problems. In his early paper “How I Do It,” Crysdale mentions that “ranula formation has been the only complication of significance.”

Consequently Crysdale advocated automatic removal of the sublingual glands [16], and this has become standard practice. In this small series of paediatric patients with severe sialorrhea treated surgically, the majority had bilateral submandibular duct relocations with concurrent removal of sublingual glands. Unexpected findings were the variability in the size; symmetry or the absence of sublingual glands, the absence or ectopic location of submandibular ducts (Table 3). In some individuals, the sublingual glands are extensive and presumably contribute a relatively large amount of the saliva volume. Therefore, the automatic removal of sublingual glands is logical and accounts for the absence of ranula formation in this series.

 A new development in pharmacologic treatment for drooling has been the use of Botulinum toxin injected into the salivary glands. Following the first report on its efficacy when injected into the parotid glands in an adult with motor neurone disease [21], it has been used as a temporary treatment in other adult neurological disorders and in children with cerebral palsy to control sialorrhea [23].

 In summary, pathological sialorrhea can have a major effect on life quality of individuals with neurological and intellectual disability, particularly in cerebral palsy where it is likely to be a lifelong problem. When it is severe, behavioural and systemic pharmacological treatments are usually not effective or sustainable. While “Botox” injections can control salivary flow, their effect it *temporary*. However in children, its use might be justified as a demonstration of the benefits of salivary control. Unfortunately, the earliest surgical treatments eliminated all saliva. Bilateral relocation of the submandibular ducts and removal of the submandibular glands has *evolved *into being the logical *contemporary *surgical treatment for this distressing condition [24]. In this small series, there were no significant complications, and its effectiveness (median control of “75%”) was high.

## Supplementary Material

Supplementary Video 1: Right submandibular duct relocation and removal of sublingual glands. The free submandibular duct is pulled through to the tongue base, and mucosal island sutured to the tongue base opposite the anterior tonsillar pillar.

## Figures and Tables

**Table 1:** Demographics and causes of sialorrhea in 16 children with severe sialorrhea treated surgically.

Sex	Males	11
	Females	5

Age	6–14 years	(Median 10 years)

Causes	Cerebral palsy	10 (3 epilepsy)
	Genetic syndromes	4 (2 epilepsy)
	Oligodendroma	1 (epilepsy)
	Global delay/autism	1

Previous surgery	1 male: bilateral tympanic neurectomies and unilateral chorda tympani neurectomy	

**Table 2:** Operations on 16 children with severe sialorrhea treated surgically.

Relocation of submandibular ducts	16
Concurrent removal of sublinguals	15
Later removal of sublinguals	1
Small or unilateral absence of sublinguals	3
Large sublinguals	3
Unilateral or bilateral absent submandibular ducts	3
Ectopic submandibular ducts	1

**Table 3:** Hospital stay and results in 16 children with severe sialorrhea treated surgically.

Hospital stay	1–4 (median 2) nights	
Complications	Swelling of one submandibular gland	1
	Tongue swelling	1
	Readmission (not drinking) at 9 days	1
Final efficacy (percent improvement) (Figure 3)	50–75% (median 75%)	
